# The influence of alcohol on prehospital diagnostics and therapy of injured patients

**DOI:** 10.1111/acer.70209

**Published:** 2026-01-23

**Authors:** Ramona Sturm, Jason‐Alexander Hörauf, Rolf Lefering, Borna Relja, Ingo Marzi, Nils Wagner, The TraumaRegister DGU

**Affiliations:** ^1^ Department of Trauma Surgery and Orthopedics University Hospital, Goethe‐University Frankfurt Frankfurt am Main Germany; ^2^ Institute for Research in Operative Medicine (IFOM) University Witten/Herdecke Cologne Germany; ^3^ Department of Orthopedic Trauma, Hand‐, Plastic‐, and Reconstructive Surgery, Translational and Experimental Trauma Research University Ulm Ulm Germany; ^4^ Committee on Emergency Medicine, Intensive Care and Trauma Management (NIS) of the German Trauma Society (DGU) Berlin Germany

**Keywords:** alcohol, injury severity, prehospital treatment, trauma patients

## Abstract

**Background:**

The prehospital assessment and subsequent therapeutic interventions are crucial for the optimal management of severely injured patients. Many trauma patients are alcohol‐intoxicated. Therefore, this study investigated the prehospital assessment of the injury pattern and management of alcohol‐intoxicated patients.

**Methods:**

In a retrospective matched‐pair analysis of TraumaRegister DGU® data patients from 2015 to 2018 with a blood alcohol level (BAL+) > 1.0‰ and without a blood alcohol level (BAL−: 0.0‰) were investigated, matched by age, gender, injured region, and mechanism. We evaluated injury assessment, prehospital therapy, and transport modalities.

**Results:**

A total of 6468 patients (3234 BAL−, 3234 BAL+) were included. Head injuries were common (56.9%), but BAL+ patients were significantly less often correctly assessed with regard to head (BAL−: 77.8% vs. BAL+: 74.2%) and facial (BAL−: 75.4% vs. BAL+: 70.0%, *p* < 0.001) injuries. Head and facial injuries were significantly more often improperly overdiagnosed in alcohol‐intoxicated patients (head: BAL−: 13.9% vs. BAL+: 21.4%, *p* < 0.05; face: BAL−: 19.8% vs. BAL+: 24.3%, *p* < 0.001), and the diagnosis of actual head injuries was underdiagnosed significantly more often in patients with BAL+. Alcohol‐intoxicated patients were sedated (BAL−: 64.9% vs. BAL+: 55.6%, *p* < 0.001) and intubated (BAL−: 39.0% vs. BAL+: 28.3%, *p* < 0.001) significantly less often and received significantly less fluid therapy (BAL−: 92.6% vs. BAL+: 90.3%, *p* < 0.001), catecholamines (BAL−: 12.7% vs. BAL+: 8.5%, *p* < 0.001), or tranexamic acid (BAL−: 10.3% vs. BAL+: 6.3%, *p* < 0.001). Admission of alcohol‐intoxicated patients to hospital was significantly more frequent at weekends and at night, and more frequent in regional and local trauma centers than in supraregional trauma centers.

**Conclusions:**

There were significant differences in the prehospital assessment of head injuries between alcohol‐intoxicated and nonalcohol‐intoxicated patients. Alcohol‐intoxicated patients were significantly less often correctly assessed, and alcohol‐intoxicated patients received fewer prehospital therapeutic interventions.

## Graphical abstract

This matched‐pair study of 6468 trauma patients analyzed the impact of alcohol intoxication on initial prehospital assessment and treatment. Alcohol‐intoxicated patients were less accurately assessed—especially regarding head and facial injuries—which led to both over‐ and underdiagnosis. They also received fewer interventions such as sedation, intubation, fluids, and medications. These findings highlight clinically relevant disparities in early trauma care and the need to optimize prehospital strategies.

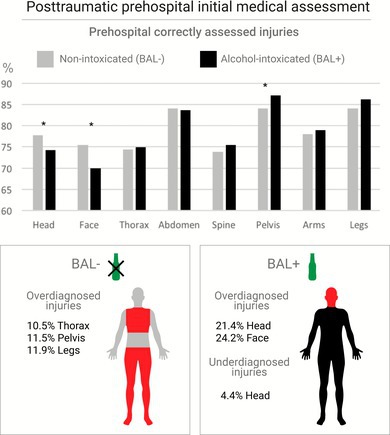

## INTRODUCTION

The treatment of trauma patients is a major prehospital and clinical challenge. Trauma is the leading cause of death in young adults (Sakran et al., 2012). Globally, injuries account for approximately 8% of all deaths, with nearly 4.4 million deaths annually (World Health Organization, 2024). The leading mechanisms of injury are road traffic accidents, self‐inflicted injuries, violence, and falls (World Health Organization, 2024). According to the data of the TraumaRegister DGU®, about 30,000 patients are admitted to participating hospitals in Germany annually (TraumaRegister DGU®, 2024). Alcohol consumption is one of the leading causes of accidents, with studies estimating that more than 25% of polytrauma patients are alcohol‐intoxicated at the time of injury (Relja et al., 2016). Regarding the causes of injuries, alcohol‐intoxicated patients are significantly more often injured due to low falls and penetrating injuries and less often injured in car and motorbike accidents (Leiblein et al., 2021). Alcohol consumption increases the financial, logistical, and personnel burden due to the need for additional laboratory tests, sonography, and cranial CT scans. This is primarily due to the reduced vigilance, more difficult anamnesis, and decreased patient compliance in the hospital (O'Keeffe et al., 2009). The management of polytraumatized patients requires a highly structured and interdisciplinary approach, involving well‐coordinated personnel, advanced diagnostic and therapeutic capabilities, and sufficient structural resources (Polytrauma Guideline Update Group, 2018).

The prehospital initial medical assessment of severely injured patients plays a crucial role in the entire treatment process. Prehospital initial medical assessment refers to the structured initial evaluation and therapy of a patient prior to hospital arrival, that is, at the accident scene and during ambulance transport. It includes the recording of vital signs, medical history, physical examination, and severity assessment in order to rapidly identify life‐threatening conditions and initiate appropriate therapy. Mortality after trauma shows a trimodal distribution: immediate death at the scene of the accident or within the first 60 min, early mortality within the first hours after trauma, and late mortality after days, which is mostly due to sepsis and multiple organ failure (Baker et al., 1980). The causes of immediate and early lethality after trauma are hemorrhagic shock (almost 40% each) and severe traumatic brain injury (Evans et al., 2010).

In Germany, trauma care is structured into local, regional, and supraregional trauma centers each equipped with specific personnel, structural, and technical resources (e.g., emergency room equipment) (Deutsche Gesellschaft für Unfallchirurgie, 2019). These centers are interconnected to ensure optimal management of severely injured patients in every region. The selection of the appropriate trauma center is based on injury pattern, severity of the injuries, patient's condition with vital signs, and logistical aspects such as transport distance, and requires a correct prehospital initial medical assessment of the patient. Accordingly, minor injuries are treated at local trauma centers, while severely polytraumatized patients are transported to supraregional centers.

For prehospital trauma care, the Prehospital Trauma Life Support (PHTLS) concept provides a structured approach to treat patients with serious injuries as quickly and efficiently as possible (Frank et al., 2014). It follows the Airway, Breathing, Circulation, Disability, Exposure (ABCDE) approach to facilitate rapid assessment and stabilization of the patient. The Glasgow Coma Scale (GCS) is widely used for assessing the patient's level of consciousness, with the areas of eye opening, verbal response, and motor response. If neurological symptoms are already present in a severe traumatic brain injury, rapid neurosurgical intervention is essential to improve the prognosis and outcome (Kühne et al., 2013).

However, the prehospital initial medical assessment of alcohol‐intoxicated patients is often more difficult. Depending on the blood alcohol concentration and patient‐specific factors, various physiological and neurological alterations occur. Alcohol intoxication leads to cognitive impairment, making it difficult to assess neurological status and complicating diagnostic procedures and treatments. Alcohol consumption also reduces the perception of pain, which together makes it more difficult to assess the severity of the injury. Furthermore, patients with alcohol intoxication show behavioural abnormalities that can range from incompliance and aggression to complete apathy (Mirijello et al., 2023). There are numerous other examples of difficult prehospital initial medical assessments of alcohol‐intoxicated injured patients, such as respiratory depression, which could be due to alcohol or chest trauma, or hypotension, which could be due to alcohol or caused by hemorrhagic shock. There are also numerous pathophysiological effects of alcohol that increase the risk of aspiration, such as cytotoxic effects on the mucosa, increased gastric acid production, or a reduction in the muscle tone of the lower esophageal sphincter (Vagts et al., 2003).

**FIGURE 1:**
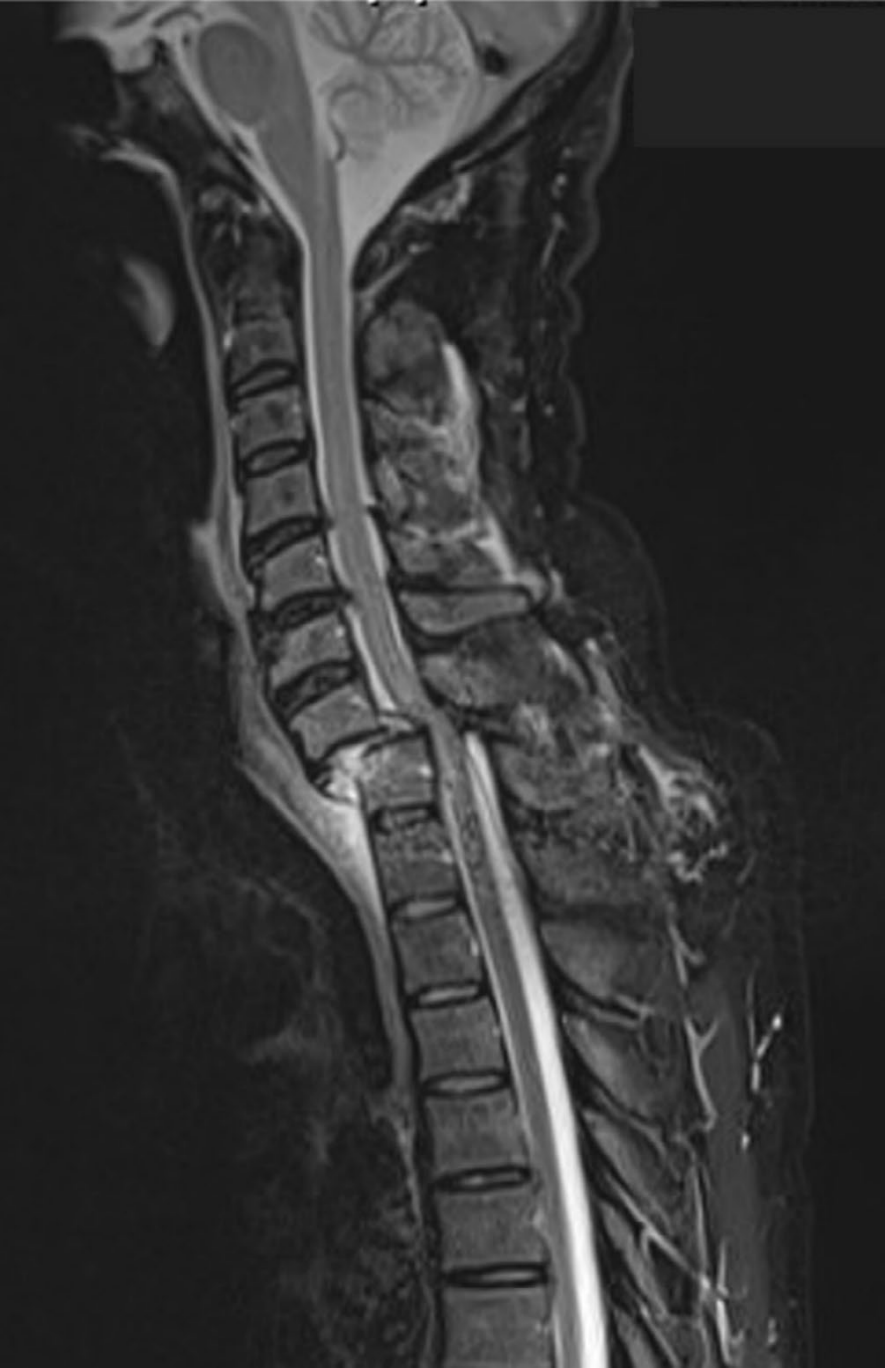
Magnetic resonance imaging of the cervical and upper thoracic spine of the case report. Dislocation of cervical vertebral body 7 against thoracic vertebral body 1 with resulting spinal canal stenosis and severe spinal cord compression.

The complexity of correct prehospital initial medical assessment in alcohol‐intoxicated patients is illustrated by the following case: A 53‐year‐old male patient with obvious alcohol intoxication was found by passersby, lying slightly near a staircase at a railway station. Communication with emergency medical services was difficult due to a language barrier and alcohol intoxication. The patient was unable to provide details about the accident but reported that he could not move his legs. The patient did not report any pain during the examination and was transported to the hospital without immobilization. Upon arrival at the hospital, clinical examination revealed paralysis of both legs, although the patient was only partially responsive due to impaired vigilance. CT imaging revealed a C7/Th1 dislocation and MRI diagnostics showed severe spinal cord compression (Figure 1). Emergency surgery was performed, involving open reduction from dorsal and dorsal instrumentation with an interne fixator (C6/7 to Th1/2).

During hospitalization, the patient later reported that he had stumbled and fallen down the stairs. Upon admission to the emergency department, the patient had a blood alcohol level of 2 per mille.

This leads to the hypothesis that alcohol consumption leads to an underdiagnosis of injury severity in the prehospital setting. The aim of this study was to determine whether alcohol‐intoxicated patients are incorrectly assessed prehospitally, whether they receive different prehospital therapeutic interventions, and whether these differences impact patient mortality.

## MATERIALS AND METHODS

Depending on their specialist expertise and the specific personnel, structural, and equipment resources, a distinction is made between local, regional, and supraregional trauma centers.

Local trauma centers have their main function in the comprehensive care of mono‐injuries and the initial treatment of severely injured patients. Regional trauma centers have the task of providing comprehensive emergency and definitive care with the corresponding surgical and intensive care capacities. Whereas supraregional trauma centers are responsible for the comprehensive treatment of all multiple and severely injured patients with constant availability of all specialist disciplines, as well as surgical and intensive care capacities.

This retrospective study was performed using data from the TraumaRegister DGU® of the German Trauma Society (Deutsche Gesellschaft für Unfallchirurgie, DGU). This registry was founded in 1993. The aim of this multicenter database is a pseudonymized and standardized documentation of severely injured patients. Data are collected prospectively in four consecutive time phases from the site of the accident until discharge from hospital: (A) prehospital phase, (B) emergency room and initial surgery, (C) intensive care unit, and (D) discharge. The documentation includes detailed information on demographics, injury patterns, comorbidities, pre‐ and in‐hospital management, course in the intensive care unit, relevant laboratory findings including data on transfusion and outcome of each individual. The inclusion criterion is admission to hospital via emergency room with subsequent ICU/ICM care or reaching the hospital with vital signs and dying before admission to ICU. The infrastructure for documentation, data management, and data analysis is provided by AUC—Academy for Trauma Surgery (AUC—Akademie der Unfallchirurgie GmbH), a company affiliated with the German Trauma Society. The scientific leadership is provided by the Committee on Emergency Medicine, Intensive Care and Trauma Management (NIS) of the German Trauma Society. The participating hospitals submit their data pseudonymized into a central database via a web‐based application. Scientific data analysis is approved according to a peer review procedure laid down in the publication guidelines of TR‐DGU. The participating hospitals are primarily located in Germany (90%), but a rising number of hospitals from other countries contribute data as well (at the moment from Austria, Belgium, Finland, Luxembourg, Slovenia, Switzerland, the Netherlands, and the United Arab Emirates). Currently, around 35,000 cases from nearly 700 hospitals are entered into the database per year. Participation in TR‐DGU is voluntary. For hospitals associated with TraumaNetzwerk DGU®, however, the entry of at least a basic dataset is obligatory for reasons of quality assurance. In addition to the basic dataset, there is a standard dataset with about 100 parameters per patient.

The present manuscript is registered under the TR‐DGU Project‐ID 2019‐043 and has been approved in accordance with the publication guidelines of the TraumaRegister DGU®.

### Patients

The measured blood alcohol level in *μ*mol/L or mg/dL was included in the dataset of the TR‐DGU in 2015 and transformed into per mille. The documentation of the alcohol level is not mandatory, and it is documented in the registry only if it was tested in routine care. The standard TR‐DGU dataset was used for the present retrospective analysis; the reduced basic dataset does not contain alcohol levels. All severely injured patients aged 16 years or more primarily admitted to a German hospital in the years 2015–2018 who had a documented alcohol level were considered. Patients with a blood alcohol level of >0 and <1 per mille were excluded. Patients admitted from other hospitals (transfer in) were excluded due to missing initial emergency room data, as well as patients without documentation of prehospital interventions. Within these eligible patients, a matched‐pair analysis was performed where the pairs consisted of patients with a zero blood alcohol level and patients with a blood alcohol level above 1 per mille. Matching criteria were age (4 groups 16–59, 60–69, 70–79, 80+), sex, injury pattern according to the Abbreviated Injury Score (AIS ≥ 3 or <3 in the body regions head, thorax, abdomen, and extremities/pelvis), and the mechanism of accident (traffic accidents by car, motorcycle, bicycle, pedestrian; high fall [>3 m], low fall [<3 m], blunt hit, gunshot, stabbing, and others).

The time of the accident, transport modalities, and prehospital interventions were analyzed. The statistical evaluation of the assessment of injury severity was carried out by means of cross‐tabulations using the clinically/radiologically diagnosed moderate injuries (i.e., AIS ≥ 2) of the individual body regions (head/face/thorax/abdomen/spine/arms/legs/pelvis) and the prehospital suspicions of the injury pattern according to the documentation of the prehospital emergency physician on scene. In the standard dataset version 2015 of the TraumaRegister DGU® used for this analysis, each body region (head/face/thorax/abdomen/spine/pelvis/upper extremity/lower extremity/soft tissue) was categorized according to the presence and severity of injury (none/minor/moderate/severe). A correct initial medical assessment of a body region was assumed when a clinically confirmed injury with AIS ≥ 2 was also documented in the prehospital form as moderate or severe, or when an injury with AIS < 2 was recorded as none or minor. Accordingly, the term “overdiagnosed” is used when a moderate/severe injury was suspected prehospitally but was not confirmed upon clinical or radiological examination. Conversely, the term “underdiagnosed” is used when a moderate/severe injury (AIS ≥ 2) was identified clinically/radiologically but was not documented as such in the prehospital form (i.e., recorded as none or minor).

The Revised Injury Severity Classification Score, Version II (RISC II), a validated and established prognostic tool, was used in this study. The RISC II incorporates 15 different variables with corresponding weights: worst injury, second worst injury, head injury, pupil size and light reaction, sex, age, preinjury ASA, penetrating trauma, hemoglobin, INR, base deficit, CPR, blood pressure, and motor response from the Glasgow Coma Scale (Lefering et al., 2014). A multivariate logistic regression analysis was performed with mortality as the outcome variable. The analysis included RISC II, the hospital care level (Level 1 trauma center), and blood alcohol concentration (BAC: positive vs. negative).

### Statistical analysis

Statistical analysis was performed using the SPSS statistical software (version 26; IBM Inc., Armonk, NY, USA). Data are given as mean with standard deviation (SD) for continuous measurements, and counts with percentages for categorical variables. The Mann–Whitney *U*‐test and Pearson's Chi^2^ test were used to compare the differences between the groups, respectively. The level of significance was assumed with a *p*‐value below 0.05.

## RESULTS

**FIGURE 2:**
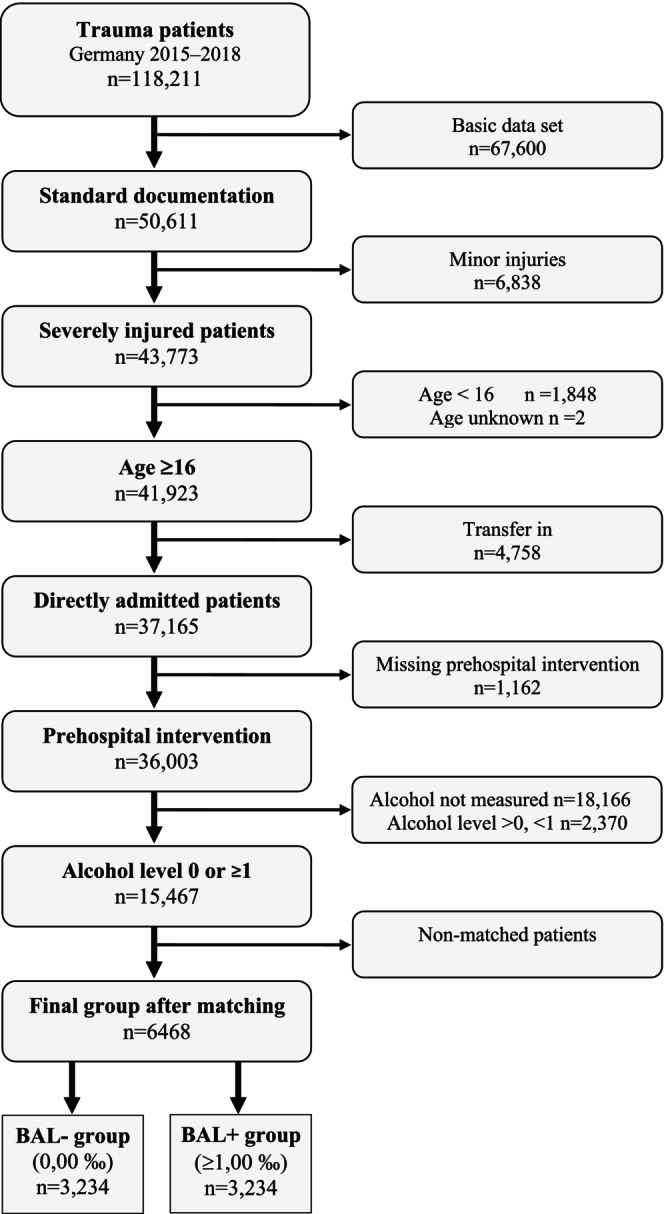
Diagram about included and excluded patients. BAL− group, negative blood alcohol level; BAL+ group, blood alcohol level ≥1‰. The flow diagram illustrates the inclusion and exclusion criteria. Severely injured patients from the years 2015–2018 were included if the standard TR‐DGU documentation form was used. Exclusion criteria were age < 16 years, secondary transfers from other hospitals, missing data on prehospital treatment, missing BAC values, or intermediate alcohol levels (>0 and <1‰). 18,166 patients were excluded because of missing BAC values. After matching for age, sex, regional injury severity (AIS), and mechanism of injury, 6468 patients remained—equally distributed between those with a BAC of 0.00‰ and those with ≥1.00‰.

Among 15,467 eligible patients, 3234 pairs of patients could be identified where one patient had a blood alcohol level of 0.00‰ (BAL−) and the other patient had a blood alcohol level of 1‰ or more (BAL+) (Figure 2). The mean blood alcohol concentration in the BAL+ group was 2.16‰. Table 1 summarizes the patient characteristics. In both groups, the patients were predominantly males (82.5%). The nonalcohol group had a mean ISS of 18.8 and a mean age of 47 years. The alcohol group had a mean ISS of 17.8 and a mean age of 46 years. Looking at the injury pattern of the individual body regions depending on Abbreviated Injury Score ≥2, no differences exist between the two groups for the regions head, face, thorax, abdomen, legs, and pelvis (Table 1). Among all patients, injuries to the head were leading (56.9%), followed by injuries to the thorax (42.1%) and spine (29.0%). Facial injuries were present in 16.9% (Table 1).

**TABLE 1:** Summary of patient characteristics.

Patient characteristics	BAL− (*n* = 3234)	BAL+ (*n* = 3234)	*p*‐Value
Male sex (*n*, %)	2668 (82.5%)	2668 (82.5%)	
Age (years ± SD)	47.4 ± 18.5	46.8 ± 18.1	0.19
BAC (‰, mean ± SD)	0.00	2.16 ± 0.78	
ISS (points ± SD)	18.8 ± 12.7	17.8 ± 11.5	0.036
Injured body regions (AIS ≥ 2)			
Head (*n*, %)	1824 (56.4%)	1856 (57.4%)	0.42
Face (*n*, %)	526 (16.3%)	565 (17.5%)	0.19
Chest (*n*, %)	1371 (42.4%)	1352 (41.8%)	0.63
Abdomen (*n*, %)	388 (12.0%)	407 (12.6%)	0.47
Spine (*n*, %)	1008 (31.2%)	867 (26.8%)	<0.001
Arms (*n*, %)	888 (27.5%)	788 (24.4%)	<0.005
Legs (*n*, %)	623 (19.3%)	592 (18.3%)	0.32
Pelvis (*n*, %)	384 (11.9%)	347 (10.7%)	0.15

*Note*: Data are presented as mean ± standard deviation (SD) or *n* (%); *p*‐value for BAL+ versus BAL− group.

Abbreviations: AIS, Abbreviated Injury Scale; BAC, blood alcohol concentration; BAL− group, negative blood alcohol level; BAL+ group, blood alcohol level ≥1.0‰; ISS, Injury Severity Score.

With the same prevalence of injury to the head, a decrease in consciousness with a GCS ≤ 8 was documented significantly more often in nonalcohol‐intoxicated patients (BAL−: 35.1% vs. BAL+: 26.8%, *p* < 0.001) (Table 2).

**TABLE 2:** Summary prehospital therapy.

Prehospital interventions	BAL−	BAL+	*p*‐Value
Intubation (*n*, %)	1259 (39.0%)	916 (28.3%)	<0.001
Catecholamines (*n*, %)	409 (12.7%)	275 (8.5%)	<0.001
Chest drain (*n*, %)	140 (4.3%)	63 (1.9%)	<0.001
Reanimation (*n*, %)	159 (4.9%)	100 (3.1%)	<0.001
Sedation (*n*, %)	2096 (64.9%)	1797 (55.6%)	<0.001
Fluid administration (*n*, %)	2851 (92.6%)	2776 (90.3%)	0.001
Pelvic binder (*n*, %)	377 (11.7%)	229 (7.1%)	<0.001
Tranexamic acid (*n*, %)	333 (10.3%)	204 (6.3%)	<0.001
GCS ≤ 8 (*n*, %)	1087 (35.1%)	834 (26.8%)	<0.001

*Note*: Data are presented as *n* and (%); *p*‐value for BAL+ versus BAL− group.

Abbreviations: BAL− group, negative blood alcohol level; BAL+ group, blood alcohol level ≥1.0‰; GCS, Glasgow Coma Scale.

**FIGURE 3:**
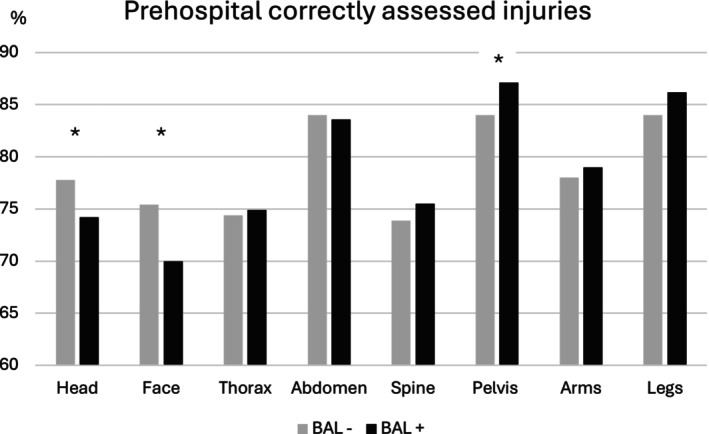
Prehospital correctly assessed injuries. The percentages of prehospitally correctly assessed injuries of the individual body regions are shown. BAL− group, negative blood alcohol level; BAL+ group, blood alcohol level ≥1‰. *p < 0.001.

**FIGURE 4:**
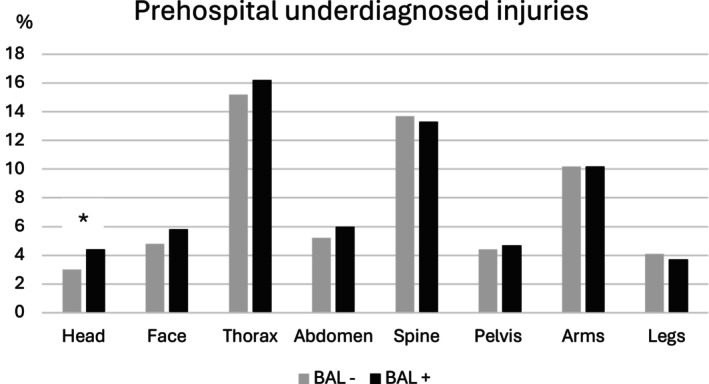
Prehospital underdiagnosed injuries. The percentages of prehospitally underdiagnosed injuries of the individual body regions are shown. BAL− group, negative blood alcohol level; BAL+ group, blood alcohol level ≥1‰. *p = 0.005.

**FIGURE 5:**
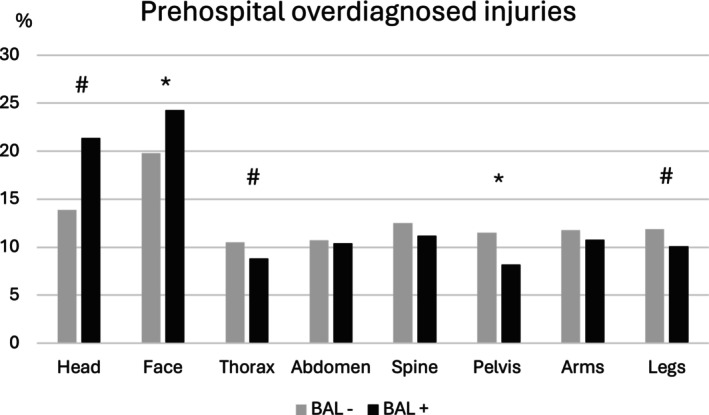
Prehospital overdiagnosed injuries. The percentages of prehospitally overdiagnosed injuries of the individual body regions are shown. BAL− group, negative blood alcohol level; BAL+ group, blood alcohol level ≥1‰. *p = 0.001, ^#^ *p* < 0.05.

In the evaluation of the prehospital initial medical assessment of the injury pattern, head injuries and facial injuries were assessed correctly significantly more often in nonalcohol‐intoxicated patients (head: BAL−: 77.8% vs. BAL+: 74.2%, face: BAL−: 75.4% vs. BAL+: 70.0%, *p* < 0.001). For injuries to the thorax, abdomen, spine, and arms, there were no significant differences in correct assessment. The injuries most frequently incorrectly assessed prehospitally are injuries to the thorax (BAL−: 15.2% vs. BAL+: 16.2%), spine (BAL−: 13.7% vs. BAL+: 13.3%), and arms (BAL−: 10.2% vs. BAL+: 10.2%), with no significant differences between alcohol‐intoxicated and nonalcohol‐intoxicated patients (Figure 3). Prehospitally, head injuries were underdiagnosed significantly more often in alcohol‐intoxicated patients (BAL−: 3.0% vs. BAL+: 4.4%, *p* = 0.005) (Figure 4). Head and facial injuries were overdiagnosed significantly more often prehospitally in alcohol‐intoxicated patients than in nonalcohol‐intoxicated patients (head: BAL−: 13.9% vs. BAL+: 21.4%, *p* < 0.001, face: BAL−: 19.8% vs. BAL+: 24.3%, *p* < 0.001) (Figure 5).

With regard to the prehospital treatments, there were significant differences between nonalcohol‐intoxicated and alcohol‐intoxicated patients with the same injury severity of the individual body regions (Table 2). Alcohol‐intoxicated patients were sedated (BAL−: 64.9% vs. BAL+: 55.6%, *p* < 0.001), intubated (BAL−: 39.0% vs. BAL+: 28.3%, *p* < 0.001), and resuscitated (BAL−: 4.9% vs. BAL+: 3.1%, *p* < 0.001) significantly less often. Alcohol‐intoxicated patients received less fluid therapy (BAL−: 92.6% vs. BAL+: 90.3%, *p* = 0.001), catecholamines (BAL−: 12.7% vs. BAL+: 8.5%, *p* < 0.001), or tranexamic acid (BAL−: 10.3% vs. BAL+: 6.3%, *p* < 0.001). The prehospital placement of a chest tube was significantly less frequent in alcohol‐intoxicated patients (BAL−: 4.3% vs. BAL+: 1.9%, *p* < 0.001). Stabilization of the pelvis by using a pelvic belt was performed significantly less often in alcohol‐intoxicated patients (BAL−: 11.7% vs. BAL+: 7.1%, *p* < 0.001).

In our matched‐pair analysis the percentage of alcohol‐intoxicated patients of all admissions to regional trauma centers (BAL−: 3.1% vs. BAL+: 18.7%) and local trauma centers (BAL−: 0.2% vs. BAL+: 2.7%) was significantly higher than that of nonalcohol‐intoxicated patients. In contrast, significantly more nonalcohol‐intoxicated patients were admitted to supraregional trauma centers (BAL−: 96.5% vs. BAL+: 78.6%). Alcohol‐intoxicated patients were more often admitted at night (BAL−: 29.7% vs. BAL+: 70.3%, *p* < 0.001) as well as on weekends (BAL−: 39.6% vs. BAL+: 51.1%, *p* < 0.001) and were less frequently transported by helicopter (BAL−: 35.8% vs. BAL+: 14.4%, *p* < 0.001) (Table 3). The expected mortality according to RISC II was 12.9% for nonalcohol‐intoxicated patients and 8.8% for alcohol‐intoxicated patients. Patients with a positive blood alcohol level showed an observed mortality of 7.4% compared with 13.3% (*p* < 0.001) in the nonalcohol group. A total of 15,467 cases were analyzed. The hospital care level (Level 1, supraregional trauma center) had minimal effect on mortality: Odds Ratio (OR) 1.02 (95% CI: 0.82–1.26). In contrast, a positive blood alcohol concentration was associated with a favorable outcome: OR 0.64 (95% CI: 0.53–0.78).

**TABLE 3:** Summary transport and outcome parameters.

Transport parameters	BAL−	BAL+	*p*‐Value
Supraregional trauma center (*n*, %)	3125 (96.7%)	2538 (78.6%)	<0.001
Regional trauma center (*n*, %)	100 (3.1%)	605 (18.7%)	
Local trauma center (*n*, %)	5 (0.2%)	88 (2.7%)	
Daytime (06:00–17:59)	2141 (66.2%)	960 (29.7%)	<0.001
Nighttime (18:00–05:59)	1093 (33.8%)	2274 (70.3%)	
Monday–Thursday	1952 (60.4%)	1386 (42.9%)	<0.001
Friday–Sunday (weekend)	1282 (39.6%)	1848 (57.1%)	
Ground transport (*n*, %)	2015 (64.2%)	2720 (85.6%)	<0.001
Air medical transport (*n*, %)	1122 (35.8%)	458 (14.4%)	
Mortality in hospital (*n*, %)	429 (13.3%)	239 (7.4%)	<0.001
RISC II (%)	12.9%	8.8%	<0.001

*Note*: Data are presented as *n* and (%); *p*‐value for BAL+ versus BAL− group.

Abbreviations: BAL− group, negative blood alcohol level; BAL+ group, blood alcohol level ≥1.0‰.

## DISCUSSION

The correct prehospital initial medical assessment of severely injured patients with potentially serious and life‐threatening injuries is a major challenge and determines subsequent prehospital therapy as well as the transport decisions and ultimately the clinical care in the appropriate hospital. In the case of alcohol‐intoxicated patients, the effect of alcohol on the patient's vigilance, the difficulty in obtaining an accurate medical history, and potential patient aggressiveness are additional complicating factors that influence the diagnosis and further treatment.

In a recent retrospective analysis of the multicenter database of the TR‐DGU by our working group, Leiblein et al. were able to show that the cause of injury differs significantly between alcohol‐intoxicated and nonalcohol‐intoxicated patients. Among alcohol‐intoxicated patients, falls from a height of less than three meters and penetrating injuries are more frequent, and traffic accidents with cars and motorbikes occur less frequently (Leiblein et al., 2021).

The initial management of the alcohol‐intoxicated polytrauma patient has a high clinical relevance, but studies on the prehospital emergency treatment of alcohol‐intoxicated patients remain scarce. The aim of this study was to investigate whether alcohol‐intoxicated patients were incorrectly assessed prehospitally and whether they received a different therapeutic intervention.

Muhm et al. investigated the prehospital initial medical assessment of injury severity by emergency physicians and found an agreement of the prehospital initial medical assessment with the ISS values recorded in the emergency department of 34%, a 36% underdiagnosis, and a 30% overestimation. Subdivided according to the injury regions, underdiagnosis of up to 47% was found in the head, thorax, abdomen, and extremities/pelvis region (Muhm et al., 2011). In contrast, our present evaluation showed that the injuries with AIS ≥ 2 were correctly assessed by the emergency physician prehospitally in over 70% depending on the body region, and overdiagnosed injuries were up to 24% and underdiagnosed injuries were a maximum of 16%. The different values could be explained by the fact that the study examined whether all relevant injuries (i.e., with an AIS ≥ 2) were recognized.

Regarding the prehospital initial medical assessment of the injury pattern, head injuries and facial injuries were assessed correctly significantly more often in nonalcohol‐intoxicated patients. Head injuries were underdiagnosed more often prehospitally in alcohol‐intoxicated patients. Head and facial injuries are more often overdiagnosed prehospitally in alcohol‐intoxicated patients than in nonalcohol‐intoxicated patients.

In both patient groups, head injuries were leading among all other injury patterns. Interestingly, the nonalcohol‐intoxicated patients were more likely to have a reduced vigilance state with a GCS of 3–8. With regard to the GCS in alcohol‐intoxicated patients, there are partly different study results (Stuke et al., 2007). Further TR‐DGU evaluations showed that alcohol‐intoxicated patients have a reduced GCS on admission to hospital (Brockamp et al., 2021; Leiblein et al., 2021). It remains to be noted that a correct assessment of the genesis of a reduced vigilance after trauma with potentially significant head injury is particularly difficult in combination with significant alcohol consumption.

Studies on the prehospital therapy of alcohol‐intoxicated polytrauma patients are scarce. The initiation of life‐saving measures, time‐critical interventions, and transport decisions is critical in determining patient outcome. It is essential to balance whether a potentially time‐consuming measure should be performed on site. Factors such as injury severity, patient condition, provider expertise, scene conditions, transport time, and potential complications of interventions must all be considered (Bernhard et al., 2015; Polytrauma Guideline Update Group, 2018).

In the present evaluation, it could be shown that, with the same injury pattern, alcohol‐intoxicated polytrauma patients receive significantly fewer prehospital therapeutic interventions. Besides sedation and intubation in case of unconsciousness or for airway protection, basic measures such as immobilization and application of a pelvic sling were also less frequently performed. Furthermore, fluid therapy or the administration of circulatory support medication with catecholamines or tranexamic acid was carried out less frequently.

Surprisingly, the lower level of prehospital treatment of alcoholized patients is not reflected in higher mortality. The observed mortality was similar to the expected mortality according to RISC II (Lefering et al., 2014). The multivariate logistic regression analysis revealed a favorable outcome in intoxicated patients, with an OR of 0.64 (95% CI: 0.53–0.78). This finding is partly explained by the inclusion of unconsciousness in RISC II, which here contributes to the favorable effect because alcohol, rather than traumatic brain injury, was more likely the cause of unconsciousness. In our working group, various studies have shown that patients with a positive blood alcohol level do not have a worse outcome compared to nonalcohol‐intoxicated patients (Leiblein et al., 2021; Wagner et al., 2019). Possible explanations may lie in the immunomodulatory influence of alcohol (Haag et al., 2022; Sturm et al., 2025).

As our study focused on prehospital treatment, the reasons for the differences in mortality cannot be determined from the present data and should be investigated in further studies. No information on education level and social status was available. Different social status and education level may have contributed to a different prehospital initial medical assessment. Social factors, in particular, should be systematically considered in the design of future studies.

Nevertheless, to prevent suboptimal care due to the poorer prehospital initial medical assessment and therapeutic intervention in alcohol‐intoxicated patients, targeted training for emergency medical services and emergency physicians is necessary.

Within the matched‐pair cohort, alcohol‐intoxicated patients were more frequently admitted to regional trauma centers and especially local trauma centers than nonalcohol‐intoxicated patients. On the one hand, it might represent the direct transport of alcohol‐intoxicated patients to the nearest hospital, possibly due to significantly less prehospital therapy. On the other hand, it might also be a result of the misinterpretation of the injury pattern of alcohol‐intoxicated patients already described. Referred to the social times of alcohol consumption, patients with a positive blood alcohol level were admitted to hospital more often in the evening and night between 6 pm and 6 am and on weekends. Riuttanen et al. determined approximately the same temporal distribution of admitted alcohol‐intoxicated patients. They showed that the relative proportion of alcohol‐intoxicated patients increases at night so that the highest rate of alcohol‐intoxicated patients was between 21:00 and 07:00 (Riuttanen et al., 2020).

The admissions at night and on weekends pose challenges for the in‐hospital management due to reduced staffing and available resources, including surgical and diagnostic capacities.

A limitation factor to consider in this study is that only the standard dataset was used, as the basic dataset does not include blood alcohol level. The vast majority of supraregional trauma centers use the standard dataset, whereas it is used less frequently by regional trauma centers and rarely by local trauma centers. Accordingly, the study mainly included patients who were admitted to supraregional trauma centers. Hence, it is not possible to provide an assessment regarding the overall distribution and selection of the respective target hospital for the general cohort of all alcohol‐intoxicated patients. In this matched‐pair analysis, the direct comparison of alcohol‐intoxicated and nonalcohol‐intoxicated patients with the same injury pattern was important. The results show that BAL+ patients were more often misdiagnosed compared to patients with BAL = 0. One possible explanation for this finding is that patients with BAL+ were discriminated against compared to BAL− patients. Another plausible explanation is the diagnostic challenge in the prehospital setting to differentiate between symptoms caused by alcohol intoxication and those resulting from physiological effects of trauma. A common example is the reduced GCS, which may be attributable either to traumatic brain injury or to alcohol intoxication. Implementing prehospital alcohol testing may support more precise differential diagnosis and therapeutic interventions.

Additionally, the retrospective design must be considered when interpreting the data, as a direct causal relationship between blood alcohol level and observed parameters cannot be proven in the retrospective analysis and the results are to be evaluated as associations. No information was available on the presence of other drugs that may influence vigilance in addition to alcohol. When interpreting the results and data, patients with missing alcohol values who have been excluded must be taken into account.

However, the advantages of this register‐based evaluation include the high case number and precise matching of patient groups based on age, sex, injury severity across different body regions, and mechanism of injury.

In conclusion, severely injured patients with a positive blood alcohol level are more frequently misjudged (moderate‐to‐severe injuries present are not recognized as such in the prehospital setting, or suspicion of moderate‐to‐severe injuries is expressed despite their absence) and receive less prehospital care. This results in a potential risk of inadequate treatment during the prehospital phase of alcohol‐intoxicated patients.

In the current study, this did not affect mortality, but this should be further investigated in a prospective study. The case report presented in Figure 1 highlights the potential severe consequences of insufficient prehospital management. The present study demonstrated prehospital insufficient treatment of intoxicated patients, which, although it did not show a negative effect on mortality in the overall collective, can have dramatic effects in individual cases.

## CONFLICT OF INTEREST STATEMENT

RL declares that a service agreement exists between the University of Witten/Herdecke and AUC (Akademie der Unfallchirurgie GmbH), which includes the statistical support in registry data analysis. The other authors declare that there is no conflict of interest.

## Data Availability

The data that support the findings of this study are available from the corresponding author upon reasonable request.
